# Connectivity of Marine Protected Areas and Its Relation with Total Kinetic Energy

**DOI:** 10.1371/journal.pone.0139601

**Published:** 2015-10-08

**Authors:** Andressa D’Agostini, Douglas Francisco Marcolino Gherardi, Luciano Ponzi Pezzi

**Affiliations:** Remote Sensing Department (DSR), National Institute for Space Research (INPE), São José dos Campos, São Paulo, Brazil; Biodiversity Research Center, Academia Sinica, TAIWAN

## Abstract

The East Continental Shelf (ECS) of Brazil is a hotspot of endemism and biodiversity of reef biota in the South Atlantic, hosting a number of Marine Protected Areas (MPAs). Connectivity of MPAs through larval dispersal influences recruitment, population dynamics, genetic structure and biogeography in coral reef ecosystems. Connectivity of protected reef ecosystem in the ECS was investigated with a hydrodynamic model (ROMS) forcing an Individual Based Model (IBM—Ichthyop), and used groupers (genus *Mycteroperca*) as functional group. The hydrodynamic output from ROMS was compared with satellite data and showed good agreement with observed surface fields. Eggs were released, in IBM experiments, from April to September along six years (2002–2007) in five MPAs along the ECS. Intrannual variability in recruitment and self-recruitment of grouper larvae was observed, as well as a negative correlation of these population parameters with total Kinetic Energy (KE) used as a metric of the physical environment. Higher KE leads to increased offshore advection of larvae, reduced total recruitment and connectivity of MPAs. Our results indicate high and uni-directional connectivity between MPAs from north to south influenced by the Brazil Current flowing in the same direction. Results also showed that some MPAs act predominantly as “sink” while others are mainly “source” areas.

## Introduction

Marine Protected Areas (MPAs) play an important role in conserving biodiversity of important marine ecosystems and should behave as a well connected network exchanging individuals between different populations [1, 2]. Connectivity among MPAs helps maintain population resilience under critical conditions (e.g. climate change) and contributes to food security through larval dispersal and adult movement supporting multi-species fisheries [3–5]. Knowledge of population connectivity and its relation with ocean circulation can greatly improve management actions for biodiversity conservation. Coral reefs are threatened habitats [6] and many reef fish populations, especially groupers (Serranidae Family, Epinephelinae Subfamily), are overexploited by fisheries [7–9]. This highlights the importance of improving our understanding of the biophysical processes controlling connectivity and coral reef population dynamics.

Connectivity of marine populations can be measured by genetic analysis (e.g. [10]), tracking natural or artificial markers in larvae (e.g. [11]), otolith chemistry (e.g. [12]) or by biophysical simulations (e.g. [13]). The biophysical modeling approach allows the integration of physical and biological processes to elicit likely recruitment scenarios and dispersion pathways [2, 13, 14]. For this purpose, lagrangian Individual Based Models (IBMs) not only help explore and compare patterns of population connectivity [15] taking into consideration the biological aspects of each individual, but also their relation to physical conditions [16]. The relevance of each physical and biological parameter to larval dispersal can be objectively tested [17, 18], allowing the identification of physical-biological interactions that lead to a higher or lower recruitment and population linkages.

The physical environment drives transport/retention of larvae during their pelagic stage and strongly influences their settlement and survival. The oceanic kinetic energy can be used as a physical descriptor of transport process, representing the energy available for displacement of a fluid parcel. For example, the influence of turbulent kinetic energy can cause larval advection offshore inhibiting larval recruitment [19]. Knowledge of how physical conditions and their persistence influence population connectivity and larval recruitment are becoming a key element in management plans for MPAs.

The East Continental Shelf (ECS) of Brazil is home of the largest coral reef complex in the South Atlantic [20] and is regarded as a center of coral biodiversity, which is now protected by a number of MPAs. Ocean circulation in the ECS is characterized by the presence of many eddies and meandering currents, showing a strong seasonal variability [21]. This complex regional circulation is likely to exert a significant influence on retention and transport of eggs and larvae between MPAs.

The present work investigates the influence of ocean circulation on the connectivity of MPAs along at the ECS. To achieve this goal an important component of reef ecosystem in the ECS, the reef fish genus *Mycteroperca* (Family Serranidae, subfamily Epinephelinae) [22–24], is used as a functional group. The trajectories of early life stages (egg and larvae) of this genus are tracked using an IBM, and larval dispersal is analyzed. The recruitment of individuals is also used to understand whether MPAs acts as source and/or sink of individuals.

This paper is organized as follows: the Materials and methods section, introduces the physical characteristics of the study area, reproductive aspects of *Mycteroperca*, description of models experiments and statistical techniques used. The Results and Discussion section, combines the presentation of our main results that are discussed with emphasis on the mechanisms of larval dispersion and MPA connectivity. The hydrodynamic model realizations are compared with satellite data, and the biophysical model results are analyzed in the context of ocean circulation to discuss recruitment and connectivity of MPAs. Finally, in the Conclusion section we present a synthesis of our main findings.

## Materials and Methods

### Study area and distribution of MPAs

**Fig 1:**
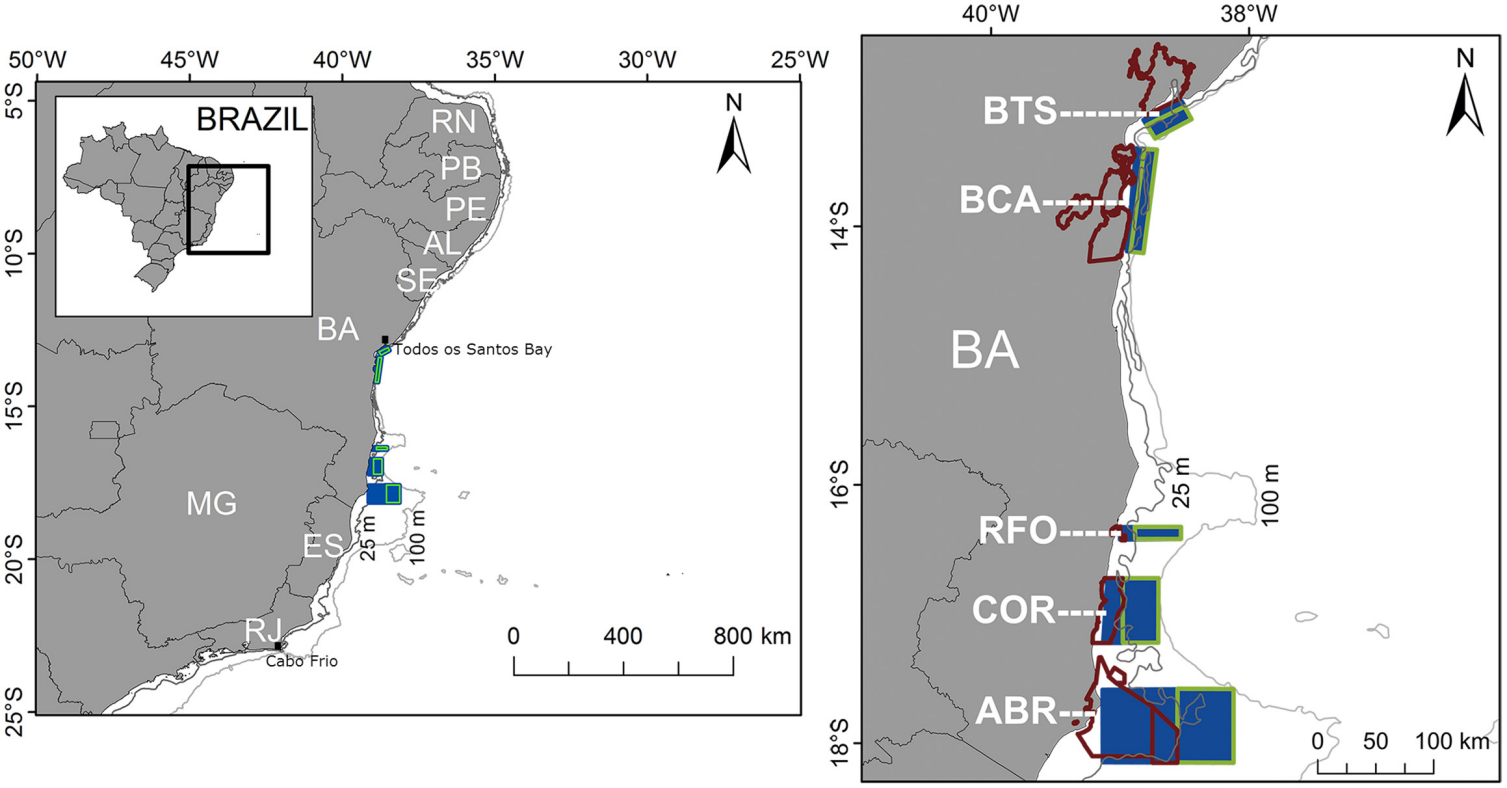
Study area map with the domain of the hydrodynamic model on the left map. The right map presents with detail the spawning areas (polygons outlined in light green), recruitment areas (polygons in navy blue), and MPAs shapes (polygons outlined in dark red). ABR = Marine National Park of Abrolhos and the Area of Environmental Protection (EPA) of Ponta da Baleia; COR = Marine Extractive Reserve of Corumbau; RFO = Municipal Park of Recife de Fora; BCA = EPA of Pratigi, EPA of Tinharé Islands and EPA of Camamu Bay; BTS = EPA of Todos os Santos Bay.

The ECS of Brazil extends from Todos os Santos Bay to Cabo Frio (Fig 1) with its narrowest width of 8 km in front of Todos os Santos Bay (Salvador, BA) and maximum shelf width of 245 km at the Abrolhos Bank [25, 26]. The South Equatorial Current bifurcation (BiSEC) is positioned between 7° and 20°S (in the upper 500 m) originating two western boundary currents: the Brazil Current (BC) flowing southwards and the North Brazil Current (NBC) flowing northwards [27]. Further south, the BC interacts with a complex topography and with the opposing flow (northward) of the North Brazil Undercurrent (NBUC) resulting in intense eddy formation between 15°S and 22°S [21]. ECS climate is marked by strong seasonality, with predominantly east and northeasterly winds during the austral spring/summer and southeasterly winds during the austral winter and autumn [28]. This seasonality is caused by the meridional shift in position of the South Atlantic High (SAH) between summer and winter [29]. The El Niño and La Niña events and the seasonal displacement of the Intertropical Convergence Zone (ITCZ) also affect ECS climate, mainly surface winds and sea surface temperature (SST) [30–32].

A number of coral reefs are found in the ECS along the State of Bahia (BA) [20] and many are protected by MPAs (Fig 1). In the south of BA (~18°S) is located the Marine National Park (MNP) of Abrolhos and the Environmental Protection Area (EPA) of Ponta da Baleia, called here the ABR group. Further north (~17°S) there is the Marine Extractive Reserve (RESEX) of Corumbau, here defined as COR group. Near 16°S is located the Municipal Park of Recife de Fora, which we call the RFO group. Moving further to the North of BA (~14°S) are located the EPA of Pratigi, EPA Tinharé Islands and Camamu Bay, called the BCA group. Finally at 13°S is the EPA of Todos os Santos Bay, called the BTS group.

### Reproductive biology of the genus *Mycteroperca*

Details of the ecology and reproductive aspects of the genus *Mycteroperca* in Brazil are still limited, therefore, we have relied on data from Northern Hemisphere (NH) populations. The few existing studies (e.g. [24, 33]) found very similar reproductive characteristics between the genus *Mycteroperca* studied in Brazil, and those described for the NH.

Several species of *Mycteroperca* occur in coral reefs in Brazil, in particular *M*. *bonaci* and *M*. *venonosa* [22–24]. A study of gonadal stages frequency and gonadosomatic index indicated that *M*. *bonaci* has a multiple spawning strategy occurring between April and September [33]. The spawning of *Mycteroperca* generally occurs during the full moon along the continental shelf break, between depths of 25 and 100 meters [34–36], but a clear biological justification for this strategy is still elusive. The genus *Mycteroperca* normally presents an egg phase of two days, and a larval phase that lasts on average 43 days [37]. *Mycteroperca* larvae display high tolerance to environment variability with salinities ranging from 20 to 50 and temperatures between 20°C and 30°C [38]. However, variations within this range can potentially affect dispersal via effects on survival, development or motility rates.

### Hydrodynamic Model

The Regional Ocean Modeling System (ROMS) was run for the ECS with a grid domain extending from 5°S to 25°S and from 25°W to 50°W (Fig 1, left), configured at a horizontal resolution of 1/24° (approximately 4.5 km) and vertical discretization of 30 sigma levels. ROMS is a three-dimensional, free-surface, terrain-following numeric model which solves the Navier-Stokes equations using Reynolds mean, hydrostatic and Boussinesq approximations [39, 40].

The model was forced every six hours with long and shortwave radiation fluxes at the surface, rain precipitation, sea level pressure, relative humidity, air surface temperature and wind velocity at 10 m. These atmospheric fields were acquired from Climate Forecast System Reanalysis (CFSR, http://rda.ucar.edu/datasets/ds093.1/#!access) dataset, with a temporal resolution of six hours [41]. Open ocean boundaries and initial conditions were forced with monthly means of SST, salinity, current velocities and sea surface height (SSH) obtained from the Simple Ocean Data Assimilation (SODA, http://www.atmos.umd.edu/~ocean/data.html) [42]. In this model setup tides were not implemented. A study in the Abrolhos Bank showed that tidal currents are weaker than subinertial currents, being more important in the cross-shelf direction than along-shelf [43]. Overall, the along-shelf current variability is mostly subinertial, more energetic and significantly correlated with synoptic winds. These authors also found important subinertial variability at the shelf break that could be related to mesoscale activity of the eddy-dominated BC. We have used a 24h period for the diel (vertical) migration in our dispersion simulations that is expected to interact with the S_2_ tide (solar semi-diurnal, period of 12.00 h). Measured amplitudes of tidal currents for the S_2_ component at the Abrolhos shelf break is 0.02 m s^-1^ [43]. A possible consequence of the incorporation of tides in hydrodynamic model could be an increase in particle dispersion. However, the consequences for the recruitment and self-recruitment are likely to be non-significant because mean meridional wind-forced currents are almost an order of magnitude higher (-0.14 m s^-1^) than cross-shelf (zonal) currents.

Three experiments were run with ROMS: a spin-up, a long-term and an hourly experiment, each one serving a different objective (Table 1). Model performance was evaluated comparing seasonal averages of monthly SST obtained from ROMS with AVHRR/Pathfinder v.5 satellite data [44] (ftp://ftp.nodc.noaa.gov/pub/data.nodc/pathfinder/Version5.0/Monthly/) for the entire period of the long-term experiment (1982–2007). Sea surface currents (SSC) derived from ROMS for the period between 1993 and 2007, were compared with the Ocean Surface Current Analysis Real-time (OSCAR) product [45] (ftp://podaac-ftp.jpl.nasa.gov/allData/oscar/preview/L4/oscar_third_deg).

**Table 1:** Experiments performed in ROMS.

Experiment Name	Interval	Output	Utility
Spin-up	1980–1981	Monthly	Equilibrium of ROMS numerical solutions
Long Experiment	1982–2007	Monthly	Performance analyzes of ROMS model outputs
Hourly Experiment	2002–2007	Hourly	Physical forcing for IBM Ichthyop experiment

### Individual Based Model

The Individual Based Model (IBM) Ichthyop is a free lagrangian tool designed to study the effects of physical and biological factors, such as ocean currents, water temperature and larval behavior, on ichthyoplankton dynamics [46]. This IBM was forced with hourly ROMS outputs of ocean current velocities, temperature and salinity. Eggs were released once every month, from April to September, starting at the first day of full moon along six years (from 2002 to 2007), totaling 36 experiments (Table 2). Eggs and larvae were tracked for 45 days, corresponding to their mean plankton larval duration (PLD), biological attributes for the genus *Mycteroperca* used in all experiments are described in section ‘Reproductive biology of the genus Mycteroperca’.

**Table 2:** Summary of Ichthyop experiment.

	Ichthyop Experiment
Number of egg release in each experiment	30.000
Bathymetric depths of spawning areas	25–100 m
Tolerable temperature range	20°C < Temperature < 30°C
Vertical movement	Diel Vertical Migration (10–30 m)
Spawning Dates(first day of full moon, 36 experiments)	2002–27/apr; 26/may; 24/jun; 24/jul; 22/aug; 21/sep2003–16/apr; 16/may; 14/jun; 13/jul; 12/aug; 10/sep2004–05/apr; 04/may; 03/jun; 02/jul; 31/jul; 30/aug2005–24/apr; 23/may; 22/jun; 21/jul; 19/aug; 18/sep2006–13/apr; 13/may; 11/jun; 11/jul; 09/aug; 07/sep2007–02/apr; 02/may; 01/jun; 30/jun; 30/jul; 28/aug

For each experiment, 30,000 eggs were randomly released within a maximum depth of 30 meters, with larger MPAs releasing proportionally more particles than smaller ones (see details in Section ‘Study area and distribution of MPAs’). The number of released particles was chosen based on a sensitivity test using simulations from 10,000 to 80,000 particles, in increments of 10,000. Percentages of recruitment, self-recruitment and mortality were tested and no significant difference were found (*p*>0.05, non-parametric Kruskal-Wallis) when varying the number of particles for any parameter. We have, therefore, decided to release 30,000 particles in all experiments for optimum larvae dispersal visualization and (low) computational cost. It should be noted that this particle number is not intended to replicate a realistic scenario, but rather offer a simulated representation of potential larval dispersal pathways to evaluate the connectivity of MPAs.

Eggs were tracked for two days until hatching and larvae were tracked for 43 days, totaling 45 days for each experiment. Information on the precise location of *Mycteroperca* aggregations in the study area are not available, therefore, the spawning areas (eggs release areas) were delimited offshore of the MPAs, within their eastern boundaries with depths ranging between 25 and 100 m. Recruitment areas are onshore extensions of the spawning areas. The fact that the continental shelf is narrower to the north and wider to the south, results in different spatial configuration for the spawning and recruitment areas (Fig 1). During simulations larvae are allowed to enter and leave the recruitment area, depending on local currents. Recruitment is only computed for those larvae that remain in the recruitment area or reach it by the end of its PLD. This allows for a conservative estimation of total recruitment for all experiments.

Particle behaviors considered in the IBM experiments are: 1) mortality by lethal temperature; 2) advection and diffusive horizontal movements; 3) vertical movement of eggs due to buoyancy; and 4) vertical movement behavior of larvae. Mortality was considered when larvae advected to waters with temperature outside the known range of larval development, between 20°C and 30°C [38]. The advective horizontal movement was simulated using the Fourth order Runge Kutta scheme. Vertical movement of eggs is related to their buoyancy, derived from a vertical velocity for prolate spheroids, which is a function of their minor and major axes, gravitational force, egg and sea water density and water molecular viscosity, described in [47].

Two days after hatching, *Mycteroperca* larvae gain vertical mobility [37] and start a vertical movement defined as Diel Vertical Migration (DVM). DVM consists of a migration to the surface at night, optimizing foraging behavior, and to lower depths at day, avoiding predation [48, 49]. A detailed study carried out by [50] concluded that larvae concentrations of groupers (Epinephelini) in the Florida Straits should result in predictable accumulations at depths shallower than 25 m. We considered, in the present study, that larvae may ascend to 10 m depth at night and descend to 30 m during the day, corresponding to a migration that would not drive larvae significantly below the Ekman layer [50].

The diffusive horizontal movement is performed based on the lagrangian coefficient of horizontal diffusivity. This coefficient is responsible for introducing certain randomness to the horizontal trajectories to allow spatio-temporal variation, a detailed description can be found in [13, 51]. We have applied a turbulent dissipation rate of 10^−9^ m^2^.s^−3^, following [52], and also used in other studies [13, 51, 53].

### Larval recruitment

Self-recruitment is defined as the number of individuals that remain in the same MPA from which they were originally released, including larvae that eventually returned to the release site. Recruitment is defined here as the number of individuals that migrated to a given MPA but were originated from another MPA. Larvae advected outside the model domain ranged from 0.97% (2007) to 10.49% (2004) and were not considered in the analysis. Mortality is the sum of larvae advected to waters with a temperature different from the range considered (20°C–30°C), divided by the total number of released individuals (excluding “out of domain” larvae) (Eq 1). Self-recruitment is standardized dividing it by the sum of surviving larvae released from the same area (Eq 2), where as recruitment is divided by the sum of surviving larvae (Eq 3). These quantities are expressed in the text and graphics as percentages for ease of understanding.

Mt=∑d(∑tc−∑tadv) (1)

SelfRect,i=∑tcii(∑tci−∑tadvi−∑tdi) (2)

Rect,i=∑tcij(∑tcj−∑tadvj−∑tdj) (3)

where:

M_t_: mortality by temperature;

d: individuals advected to areas with lethal temperatures (< 20°C or > 30°C);

c: individuals in the model domain at the end of the experiment;

adv: individuals advected outside the model domain at the end of the experiment;

Rec: recruitment percentage;

Self-Rec: self-recruitment percentage;

c_ii_: individual originated from MPA “i” recruited to MPA “i”;

c_ij_: individual originated from MPA “i” recruited to MPA “j”.

After testing data for normality with a Mann-Whitney test (*p*<0.5), a nonparametric variance analysis (Kruskal-Wallis ANOVA) was performed (significance level of 5%) to test differences in recruitment and self-recruitment, considering the spawning month, year and release area (MPAs). A Transition Probability Matrix (TPM) was calculated using the number of larvae originated from MPA “i” which was recruited to MPA “j”, divided by total survival individuals. As proposed by [13], we calculated the connectance of MPAs, given as the number of nonzero connections of the TPM divided by the square size of TPM, representing the relative degree of connectivity between MPAs.

The total Kinetic Energy (KE) per unit mass is used here as a proxy for the influence of the physical environment on larval dispersal, and its linear correlation with self-recruitment, recruitment and total dispersal distance were calculated. The latter is computed from the total distance traveled by larvae during each of the 45 days experiments. Eq 4 was used to calculate the KE, which takes into account the zonal and meridional surface currents, and corresponds to the sum of the turbulent and time-mean components of the kinetic energy field. We calculated the KE from monthly ROMS surface current outputs corresponding to the period and location of larval dispersion (see Fig 1).

KEm=12*(U2+V2) (4)

where:

KE/m: total kinetic energy per unit mass (m^2^.s^-2^);

U: zonal component of current velocity (m.s^-2^);

V: meridional component of current velocity (m.s^-2^).

## Results and Discussion

### Hydrodynamic model evaluation

**Fig 2:**
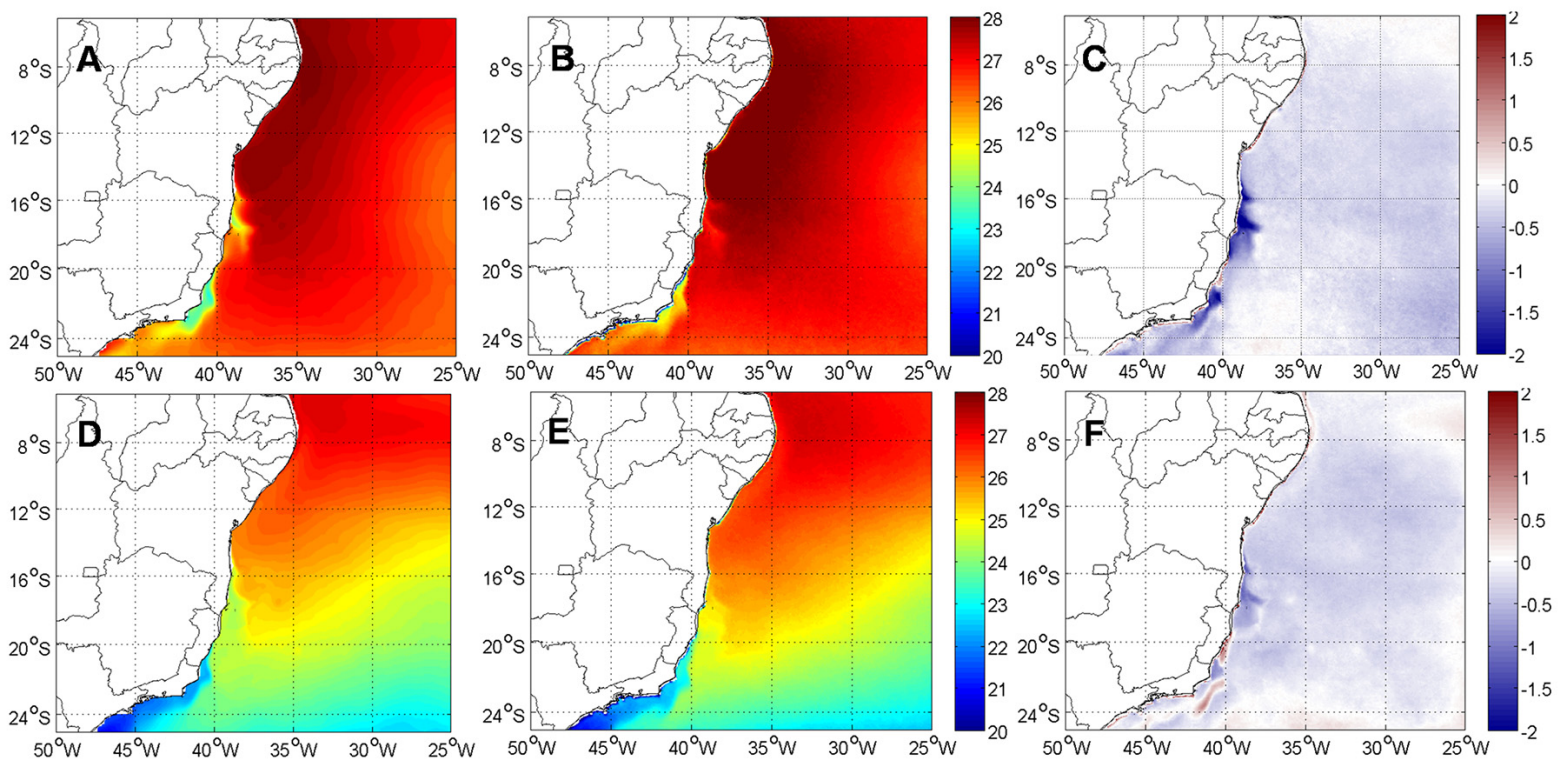
Summer and winter SST means (°C), of ROMS (summer A, winter D), AVHRR (summer B, winter E) and their difference (ROMS—AVHRR, summer C, winter F).

Comparisons between seasonal SST means (summer and winter) obtained from ROMS and AVHRR data show good agreement, indicating that simulations represent the main oceanographic features found in the ECS (Fig 2). The hydrodynamic model accurately reproduced the main oceanographic features such as cold upwelling water over the shelf (Cabo Frio upwelling at 23°S) and mesoescale eddies. Seasonal maps of SST reveal negative ROMS bias in coastal waters, caused by algorithm underestimation of AVHRR/Pathfinder v.5. [54] found similar results for the area and [51] reported the same trend in the Cabo Frio upwelling region. Discrepancies, especially in upwelling regions, may be related to a warm coastal bias up to 5°C in the AVHRR/Pathfinder v 5.0 data [55]. Summer SST difference (ROMS—AVHRR) in coastal regions are larger reaching -2°C, while in winter this negative bias drops to -1°C, particularly at the upwelling region of Cabo Frio (23°S and -42°W), Abrolhos and Royal Charlotte Banks (16° to 20°S and -39°W) (Fig 2). Water masses obtained from ROMS solutions are consistent with those described by [56] at 20°S and [51, 57] based on comparisons with is situ data collected at a nearby site (24°S).

**Fig 3:**
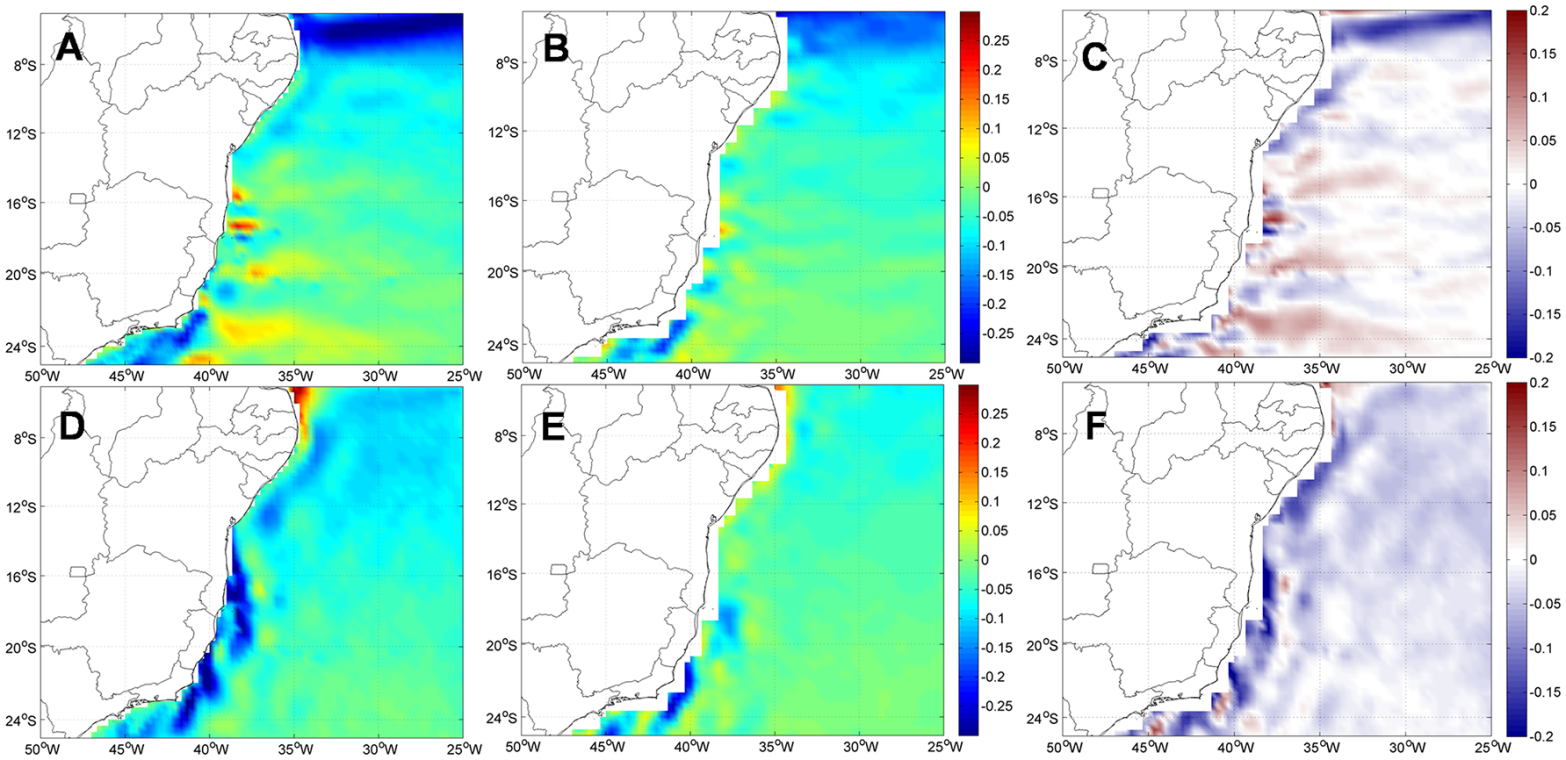
Annual SSC means, in m.s^-1^, of zonal component (U, above) and meridional component (V, below) from ROMS (A, D) and OSCAR (B, E), and their differences (ROMS—OSCAR, zonal C, meridional F).

Amplitudes of the zonal (U) and meridional (V) velocity components obtained from ROMS are comparatively more energetic than the OSCAR velocities, especially close to the coast (Fig 3). Nevertheless, current directions (Fig 3) and seasonal variability (not shown) obtained with ROMS are consistent with those from OSCAR. Summer intensification of both zonal and meridional velocities forced by stronger winds in this region [28] is evident in ROMS results. This variability is related to the SAH meridional oscillation due to seasonality of solar radiation [29].

Between 15°S and 21°S (Fig 3), higher eastward zonal currents combined with southward meridional currents, observed in both ROMS and OSCAR data, are caused by eddy activity, as described by [21]. Similarly, around 21°S and 39°W, westward zonal currents and southward meridional currents are coincident with the position of the cyclonic Vitória Eddy (VE) south of the Vitoria-Trindade Ridge [58]. ROMS zonal and meridional components are compatible with the meandering and summer intensification of the BC [28] and eddy activity in this region [21, 58]. [59] also detected intense mesoscale activity and the occurrence of cyclonic and anticyclonic eddies throughout the year using ROMS. Amplitude differences of surface currents between ROMS and OSCAR may be partially related to differences in horizontal resolution. Average (SD) zonal and meridional total current components measured from moorings in the Abrolhos Bank [43] are -0.03 (0.14) and -0.14 (0.15), respectively and compare very well with ROMS realizations shown in Fig 3.

### Annual and interannual variability of larval dispersal

**Fig 4:**
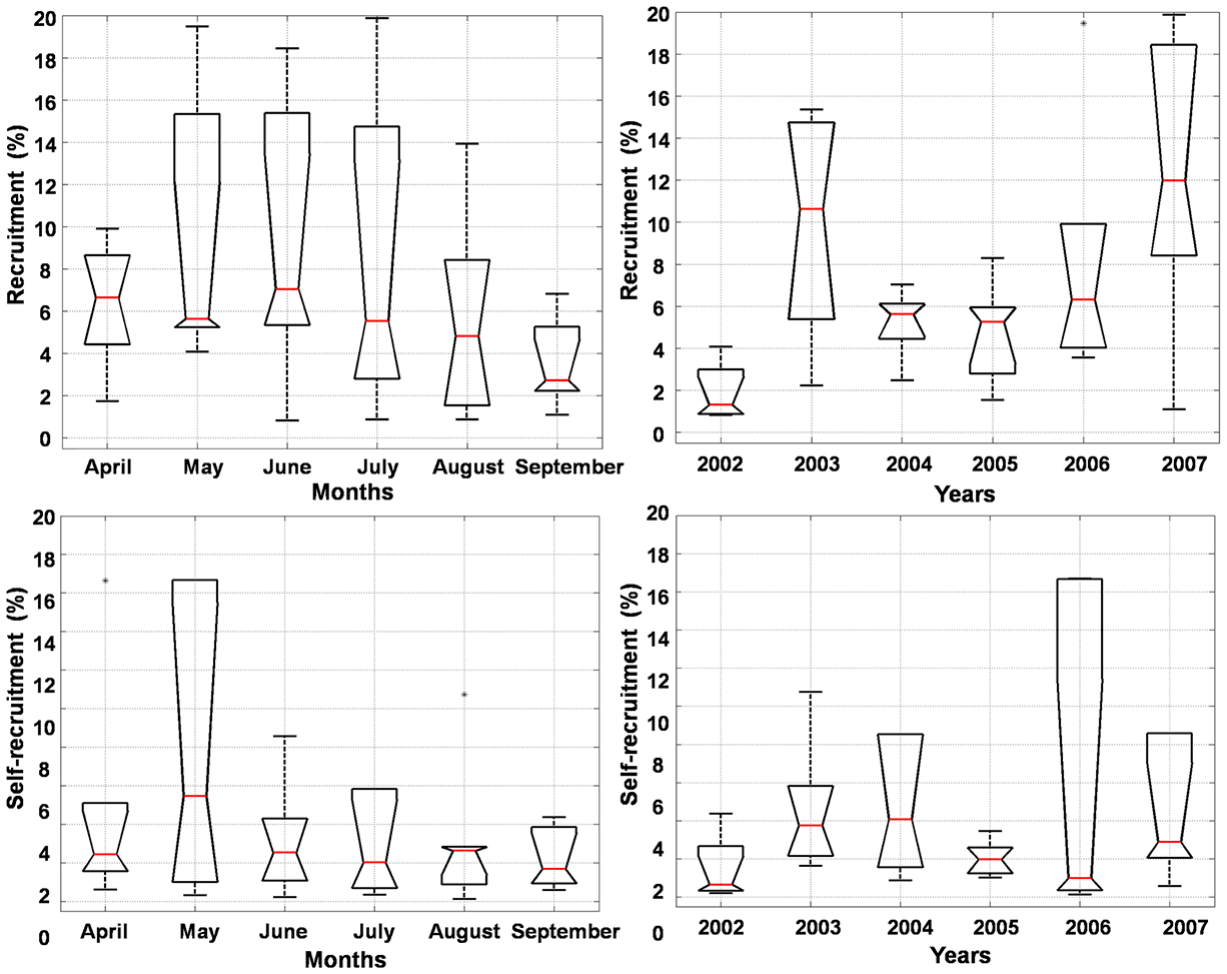
Median and quartiles of intrannual (left) and interannual (right) variability from self-recruitment (above) and recruitment (below) of *Mycteroperca* genus.

Significant differences (*p* = 0.0014) in larval mortality by lethal low temperature (<20°C) among the spawning months (April to September) were observed, with increased mortality during winter months (August and September). On the other hand, interannual (2000–2007) mortality by temperature did not differ significantly. Self-recruitment and recruitment did not present significant differences among the spawning months (*p* = 0.4489 and *p* = 0.4606, respectively), although they tend to be slightly higher from April to July compared to the period between August and September (Fig 4). Interannual self-recruitment variability is not significant (*p* = 0.546), but recruitment is significantly different (*p* = 0.0072), being lower in 2002, 2004 and 2005, and higher in 2003, 2006 and 2007 (Fig 4). Extreme years illustrate these differences with low self-recruitment and recruitment for 2002 (1.48% and 1.9%, respectively), and high figures in 2007 (10.69% for self-recruitment and 12% for recruitment). Our results point to the importance of low, but constant, larval retention for reef fish populations with little interannual variability. Interannual self-recruitment variability is not significant because percentages are very low and outlier amplitudes are large. Being a predominantly local process, self-recruitment is more susceptible to (variable) local hydrodynamics resolved by our high horizontal resolution (4.5 km) grid. Our results agree with the paradigm that biophysical retention of larvae in reef-fish populations are generally low ([60] and references therein).

**Fig 5:**
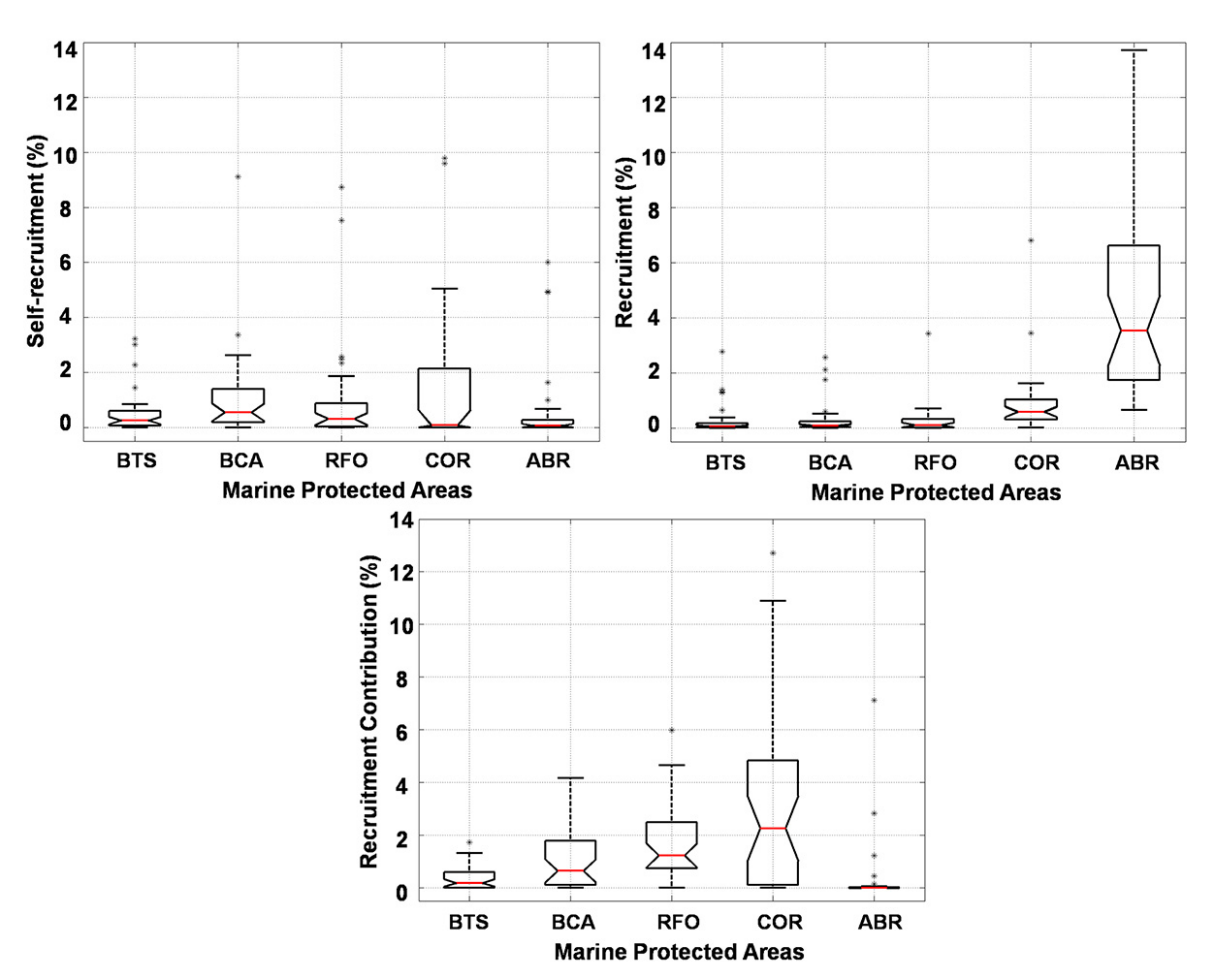
Median and quartiles of self-recruitment (left), recruitment (right) and the recruitment contribution from each MPA.

Self-recruitment, recruitment and the relative contribution of each MPA to recruitment, the latter giving the relative behavior of MPAs as source and/or sink, presented significant differences (*p* = 0.0124, *p* = 1.88e-19, *p* = 1.79e-13, respectively) (Fig 5). Self-recruitment and recruitment are similar for BTS, BCA and RFO, indicating that their dependence on larvae coming from other MPAs can be as important as self seeding. It is not possible, however, to assume that these MPAs behave primarily as sink because BCA and RFO are important sources of larvae contributing to recruitment in other MPAs. COR is a major source of larvae to other MPAs and one with the lowest self-recruitment rates, which is slightly lower than recruitment. This is an indication that COR behaves both as an important sink and source of individuals. In this complex network connected by dispersion, where reefs may assume different roles, the ABR reefs show the largest differences among recruitment (high), self-recruitment (low), and contribution to recruitment in other MPAs (very low), acting predominantly as a sink area.

**Fig 6:**
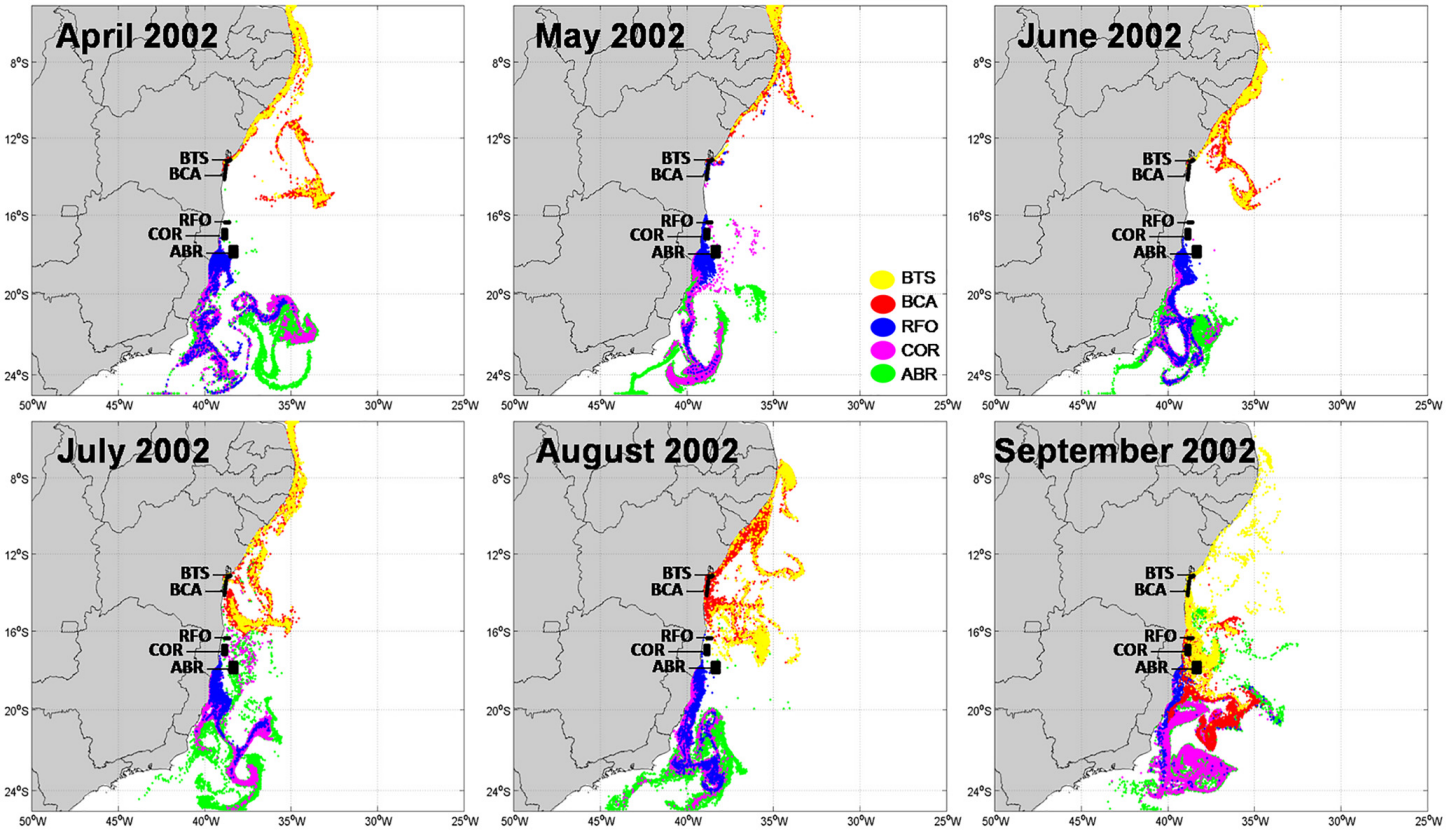
Modeled larval dispersion from April to September of 2002.

**Fig 7:**
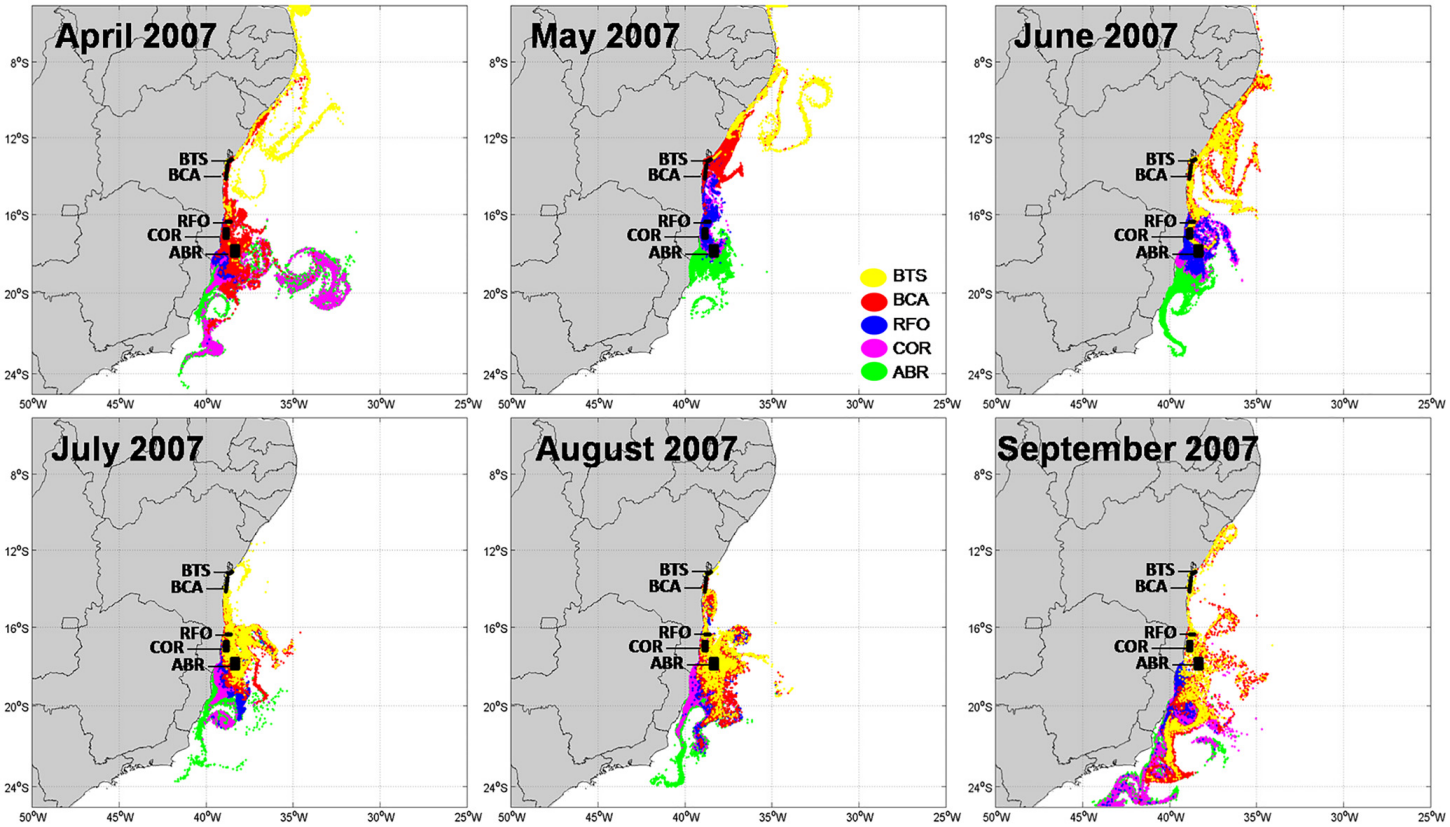
Modeled larval dispersion from April to September of 2007.

Offshore larvae advection was higher in 2002 than in 2007 (Figs 6 and 7), coincident with the lowest self-recruitment and highest recruitment percentages, respectively. The contrasting spatial aspect of larval dispersion shown in Figs 6 and 7 agrees with the numerical results described above. Between April and June, 2002 occurs a north-south separation of dispersal trajectories, when larvae spawned in the northern areas of Bahia (BTS and BCA) were transported further north, whereas those spawned in southern areas (RFO, COR and ABR) migrated southwards (Fig 6). This separation coincides with the area of the BiSEC, between 7°S and 17°S [56,61], which seems to influence the direction of larval dispersal. Thus, dispersion in the northernmost MPAs in 2002 is under the influence of the NBC transporting larvae northwards, while the BC drives larvae released in the southern MPAs further south. The separation of trajectories caused by the BiSEC is not so evident in 2007, with only some minor influence between April and June (Fig 7). It is easy to see that in both years, larvae are captured by eddies and meanders that tend to drive them offshore.

### Influence of Total Kinetic Energy in larval dispersal

**Fig 8:**
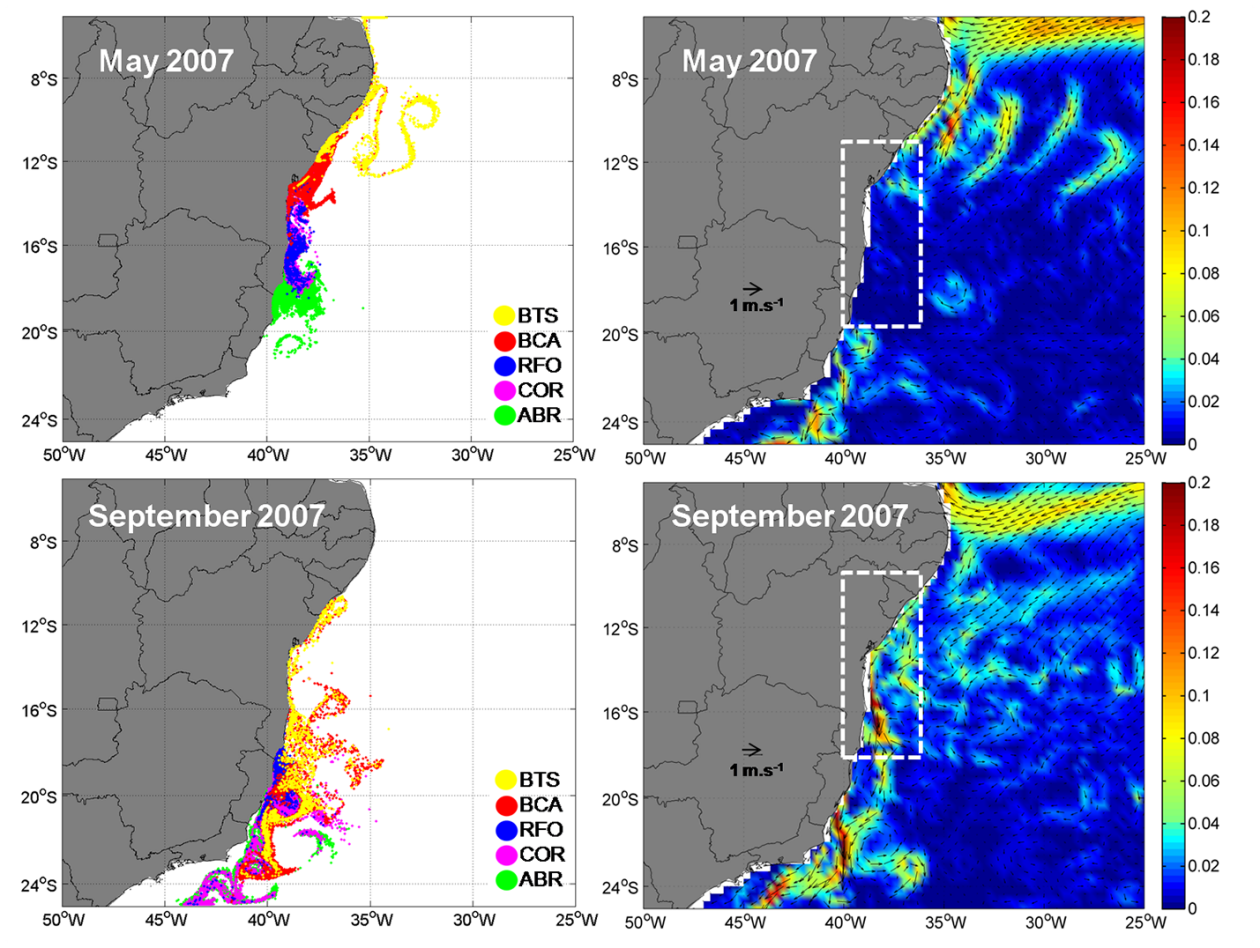
Larval dispersal (left), KE (m^2^.s^-2^, right) and surface current vectors for May (top) and September (bottom) of 2007. The dashed areas highlight the region where the spawning and recruitment areas are located.

**Fig 9:**
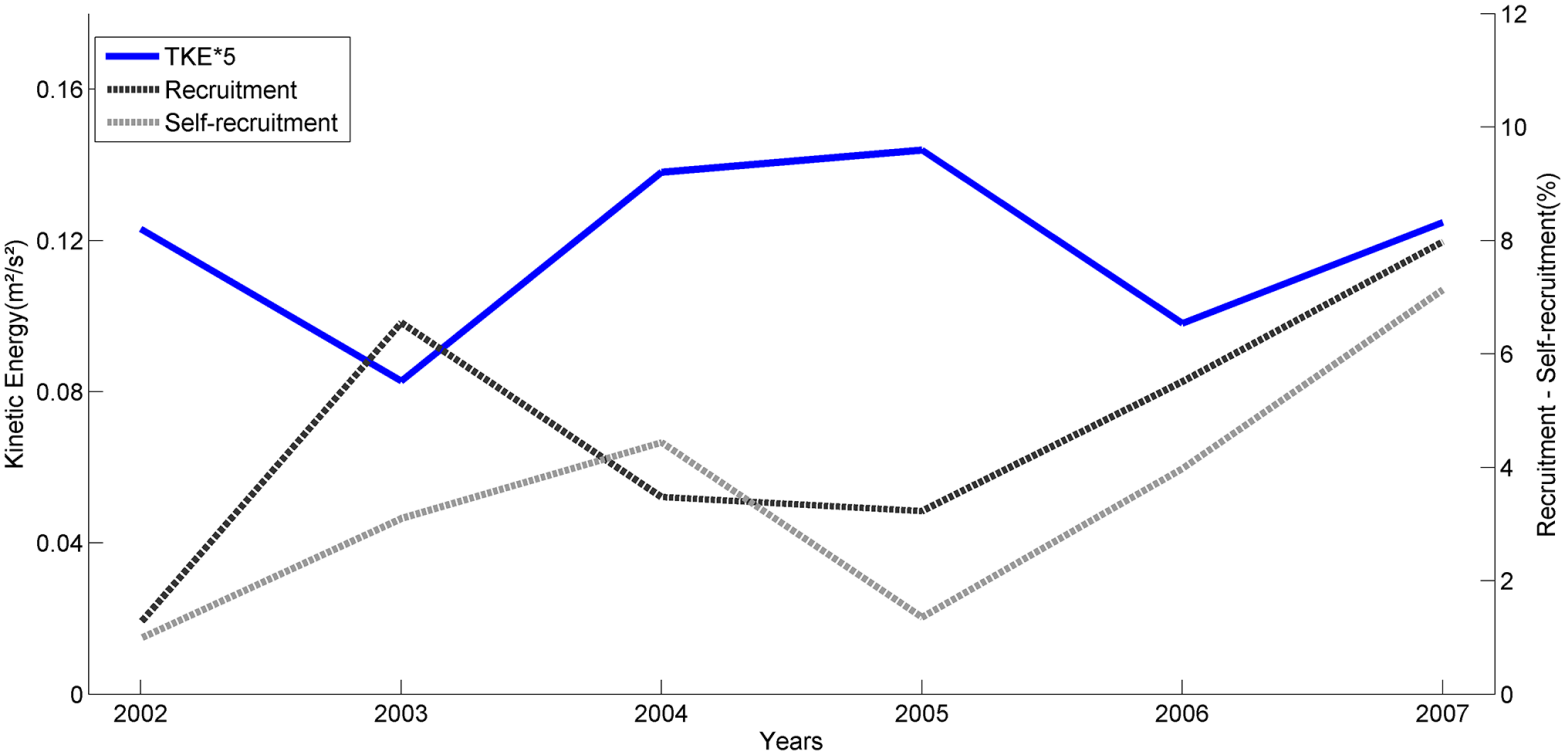
Annual means of KE (multiplied by 5 for better visualization), recruitment and self-recruitment percentages and velocity components U and V.

Our results show that recruitment is high between April and July, and tends to reduce from August to September. Extremes values of self-recruitment and recruitment percentages are found in May and September, 2007 (Fig 8). This is highlighted in the dashed area of Fig 8, where high KE in September 2007 is coincident with the lowest percentages of self-recruitment (0.6%) and recruitment (1.1%). In contrast, the observed low KE in May is coincident with the highest percentage of self-recruitment (47.15%) and recruitment (15.3%). Looking at the interannual scenario, there is a tendency for higher values of self-recruitment and recruitment during the years of lower KE (Fig 9) and vice versa. This inverse relation results in significant negative correlations of KE with mean self-recruitment (*r* = -0.34, *p* = 0.0408) and recruitment (*r* = -0.53, *p* = 0.0008). The importance of retention and dispersion of particles to the connectivity of MPAs should be assessed as relative spatio-temporal dependencies within the network. The spatial scale of energy variability influences not only the direction of larval transport, but also the relative importance of self-recruitment and recruitment for a MPA.

Mean zonal currents are predominantly eastwards, dispersing larvae away from the recruitment areas and mean meridional currents disperse particles southwards, from BTS to ABR. The KE and the mean direction of flow largely explain the highest rates of recruitment for southern MPAs (COR, ABR). The strong southward meridional component also induces an intense southward advection of larvae spawned in ABR, leading to lower self-recruitment and preventing larvae advection to MPAs located to the north.

**Fig 10:**
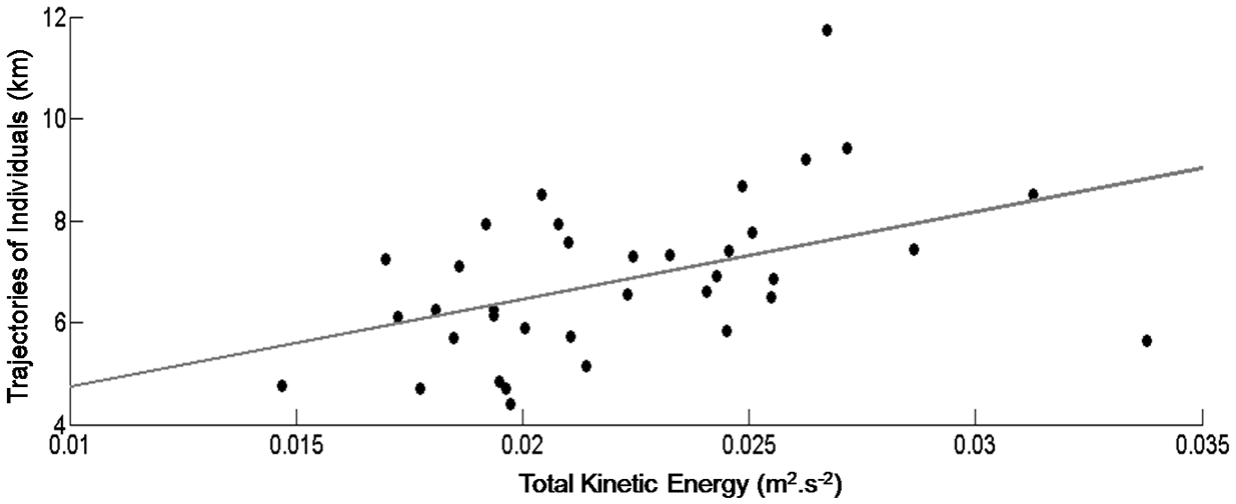
Monthly means of KE, in m^2^.s^-2^ and larvae total dispersal distance, in km.

It is expected that high KE in the region would lead to increased larval advection and distance traveled within our model domain (Fig 10). This is confirmed by a positive correlation of 0.46 (*p* = 0.0047), suggesting that total distance travelled by larvae is largely controlled by KE. [19] found similar results comparing turbulent kinetic energy and the recruitment of anchovy in Mediterranean Sea. According to these authors, when turbulent kinetic energy was higher (due to the Atlantic Jet), anchovy recruitment was considerably lower because of larval transport away from the continental shelf.

Our results indicate that ABR has a stronger dependence from individuals coming from other MPAs for population maintenance. Considering the exchange of individuals between these MPAs, their connectivity through larval dispersal has the potential to define the spatial scale of ecological interactions among fish populations [62]. Also, the spatial heterogeneity of connectivity within MPAs has important implications to the dynamics of recruitment and the persistence of local populations [63], particularly along the north-south mean flow direction.

The significant interannual differences in recruitment could be partially related to El Niño and La Niña events that occurred with low to moderate intensity between the years of 2002 and 2007. It is known that El-niño Southern Oscillation (ENSO) directly influences the climate of the ECS [30, 32, 64] representing an important source of variability in the tropical Atlantic. Positive correlations between Ninño3 and SST anomalies predominate in this region, meaning that the ECS experiences anomalous sea surface warming (cooling) during an El Ninño (La Ninña) event [64]. These authors also showed that wind stress anomalies during the El Niño are oriented southeastward, leading to a predominant offshore (northeastward) mean surface transport.

Winds, currents and mesoscale features (e.g. eddies) are known to contribute to recruitment variability [65, 66]. Our simulations produced clear indications that the BiSEC does influence larval dispersal of *Mycteroperca* in the ECS, as the result of divergent surface fluxes of the BNC (northward) and BC (southward) (see [27]). In addition, annual variability of larval dispersal seems to be related to changes in the geographical position of the BiSEC described by [61]. These authors used numerical modeling to verify that the latitudinal displacement of the BiSEC has an annual cycle as a response to seasonal changes in the magnitude of wind stress. From April to September the BiSEC moves southwards (17°S in July) from its mean position (15°S), and in November the BiSEC moves northwards (~13°S).

It has been shown that the cyclonic Tortuga gyre recirculate larvae in the Florida Keys Archipelago, promoting greater larval retention and recruitment to other sites [65]. Similarly, we observed in our simulations that some larvae are transported away from the Abrolhos and Royal Charlote banks with potential to reach other MPAs away from our model domain. Possible recruitment sites (MPAs) to the north are the EPA of Coral Reefs at Rio Grande do Norte (RN) State, and Marine State Park of Areia Vermelha located at Paraíba (PB) State. Many species of *Mycteroperca* are also found associated with rocky bottoms to the south as in Arraial do Cabo (RJ) [22, 67, 68], thus being also possible recruitment sites. Recruitment outside the model domain may occur especially with larvae spawned in ABR, submitted to strong southward advection. Eastwards of our domain, we observed larval transport towards Trindade and MartimVaz Islands (20.5°S and 29.3°W), where the occurrence of several species of *Mycteroperca* has been reported [69, 70]. So, it is possible that *Mycteroperca* populations living at the ECS and the Trindade and Martin Vaz Archipelago are connected via larval migration.

It is important to note that groupers spawn occur during full moon and this lunar reproductive periodicity may be related to the importance of tidal amplitude cycle in larval transport, representing a strategy to improve dispersion [34]. Our simulations did not include tides but, as shown elsewhere, surface currents in the Abrolhos Bank are mainly correlated with synoptic surface winds. However, it is recommended that future studies in the region include tidal forcing in the hydrodynamic model.

Other factors may also influence larval dispersal, such as the DVM behavior. When larvae migrate vertically to deeper waters, they are influenced by currents with magnitudes and directions different of that from the surface. [17, 50] showed evidences that DVM had a decisive role in larval dispersion, in addition to the local hydrodynamics. [18] also found that larvae dispersal in the Irish Sea depended strongly on DVM when compared with simulated passive larvae. These authors showed that passive larvae presented higher dispersion, being transported to outside recruitment areas, while larvae with the strategy of vertical migration presented greater retention and connectivity.

### Connectivity between MPAs

**Fig 11:**
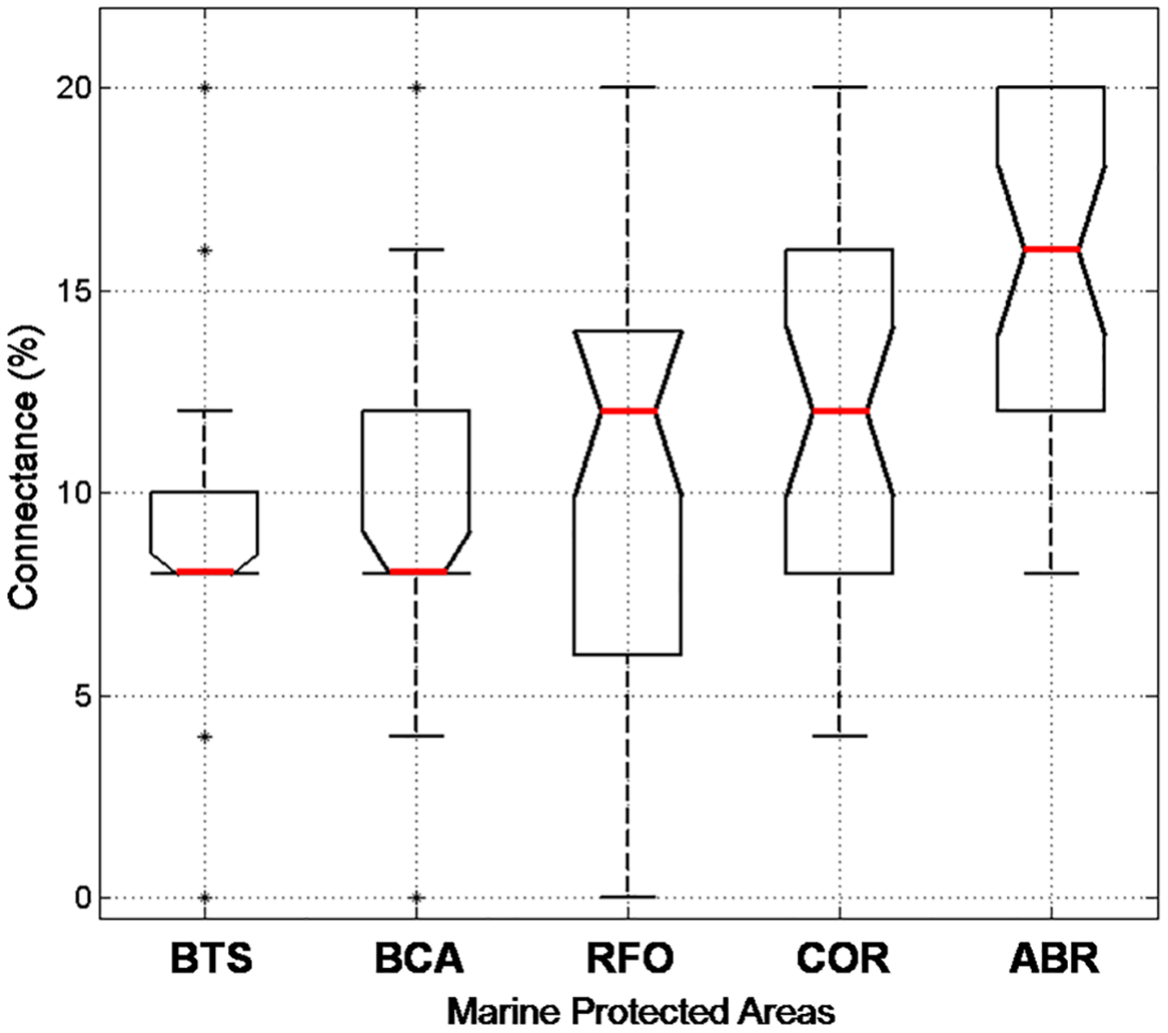
Medians and percentiles of connectance between MPAs.

The connectance parameter was 11.2%, with considerable variation between MPAs. Connectance between MPAs, calculated from a 5 x 5 matrix with 25 possible connections, presented significant interannual differences (*p* = 0.029), and strong similarity between months (*p* = 0.604). The lowest connectance was observed in 2002 (37%) and the highest in 2007 (71%), which are consistent with the modeled recruitment between MPAs (see Fig 9). An ANOVA analysis showed that connectance between MPAs was significantly different (*p* = 0.0001) with connections increasing from north to south (Fig 11). ABR presents the highest median connectance (16%) and BTS showed the lowest value of connectance (8%).

**Fig 12:**
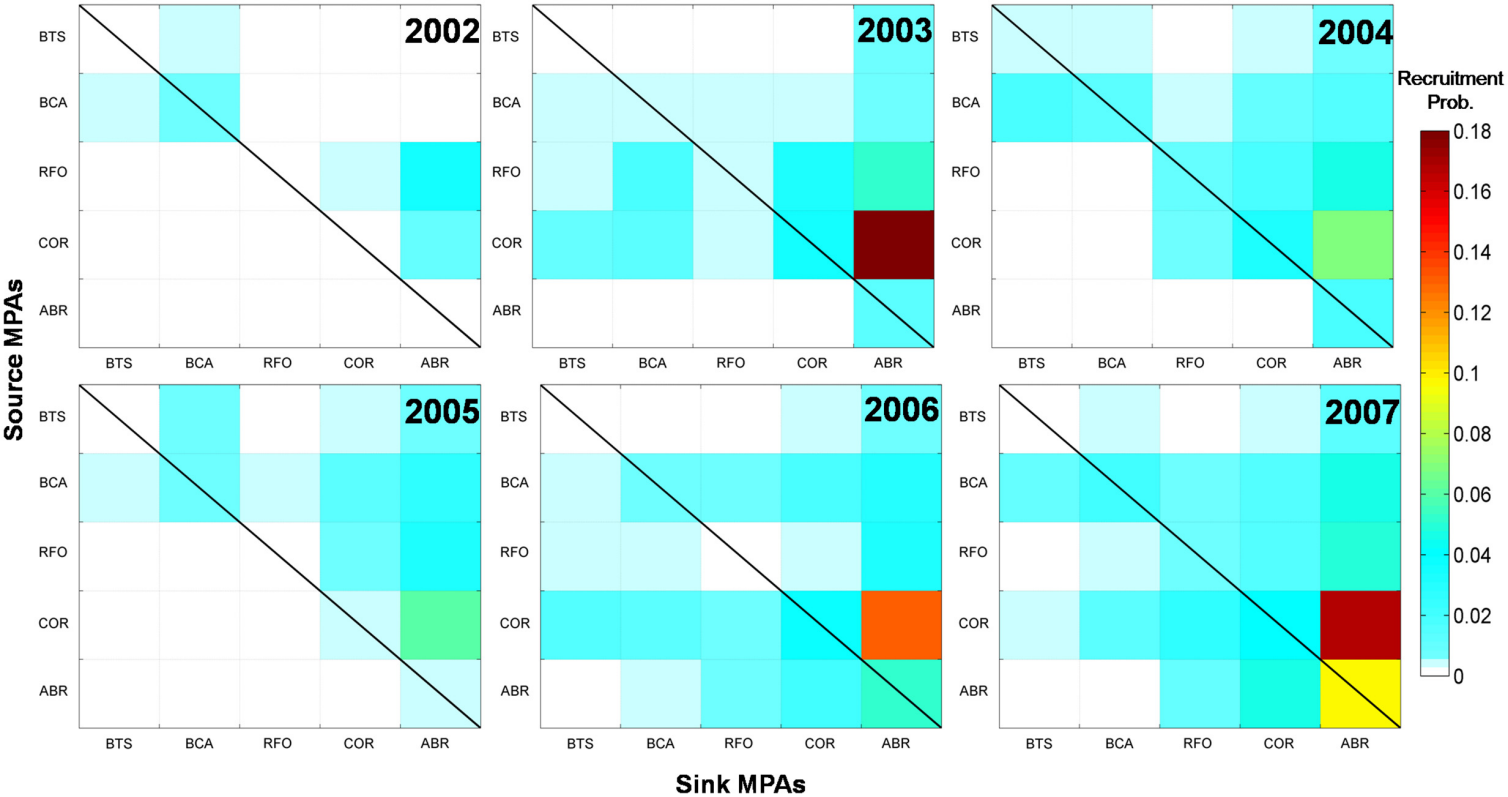
Annual Transition Probability Matrices (TPM) between spawning (source) and recruitment (sink) MPAs. The section above the black diagonal line is an upstream transport of individuals and below part represents the downstream transport. Self-recruitment is represented by the areas below the diagonal line.

The Transition Probability Matrix (TPM) indicates that migration of individuals from one MPA to another (or connectivity), occurs preferentially from north to south (downstream), with ABR being the main sink area (Fig 12). ABR receives individuals from all other MPAs, especially from the nearby RFO and COR, but its contribution to other MPAs is low. COR is the second largest sink area, and the major source area of individuals to other MPAs. Connectivity of the other three MPAs (BTS, BCA and RFO) fluctuates yearly with lower transition probabilities.

[13] analyzed the connectivity in 115 Mediterranean MPAs for the dusky grouper (*Epinephelus marginatus*) in a biophysical model and found smaller proportions of connectance (only 3–4%) than those found in the present study. Adding natural mortality to our simulations could possibly have resulted in reduced dispersal and lower connectivity. Estimates for natural mortality rates (per year) for *Epinephelus morio* adults vary from 0.16 to 0.35 [71]. On the other hand, [72] showed results of otolith analyses that estimated a pelagic larval duration of 30 to 80 days for *Epinephelus itajara*, with the potential for delayed larval metamorphosis. Adopting a longer PLD (>45 days) would most likely increase the calculated connectivities presented here. Our results should, therefore, be interpreted as conservative figures that offer a balanced scenario of connectivity, from which more detailed experiments (*in situ* or *in silico*) can be devised.

Our connectance and TPM analyses highlighted a strongly uni-directional connectivity of MPAs. To our knowledge, the present work offers an original insight into the connectivity of MPAs in the Brazilian ECS. Understanding the dispersal and recruitment in reef environments helps evaluate management approaches to marine conservation as these MPAS represent favorable environments for the development of individuals for the recovery of exploited fish populations. [73] suggested the increase of twice the density, approximately three times the biomass, and an increase of 20% to 30% in body size and diversity of organisms in marine reserves, when compared to unprotected areas.

The connectivity among local populations serves as a sound basis for the creation and improvement of MPAs networks as it takes into consideration the population dynamics within and among MPAs. For an effective functioning of such networks, each MPA must be self-sustaining or adequately connected with other MPAs via larval dispersal, thereby ensuring the effective conservation of marine biodiversity [2]. Future connectivity studies along the Brazilian coast and oceanic islands should consider using a larger dataset of reef-fish species and detailed information of their reproductive biology, as carried out by [14] for the Caribbean region.

## Conclusion

The present work investigated the connectivity of MPAs at the ECS of Brazil and its relation with KE through biophysical modeling. We showed that KE is a convenient descriptor of how the physical environment influences larval dispersion, incorporating important characteristics of the mesoscale velocity field. This is a pioneer work aimed to comprehend how coral reef MPAs are connected through larval dispersal of the genus *Mycteroperca*. Compared to other published results, the connectivity is high and occurs mainly from north to south, due to the BC. Mesoscale hydrodynamic regimes also exert a strong influence on the connectivity of MPAs. When KE is high, larvae are advected out of continental shelf, thus decreasing recruitment and connectivity among MPAs. The contribution of each MPA for recruitment differs, with some MPAs acting as source of individuals and/or sink. Connectivity can contribute to the replacement of individuals in reef environments, especially of commercially exploited fishes such as groupers.
